# Antidepressant use and risk of adverse outcomes: population-based cohort study

**DOI:** 10.1192/bjo.2022.563

**Published:** 2022-09-13

**Authors:** Narinder Bansal, Mohammed Hudda, Rupert A. Payne, Daniel J. Smith, David Kessler, Nicola Wiles

**Affiliations:** Department of Population Health Sciences, Bristol Medical School, University of Bristol, UK; Population Health Research Institute, St George's, University of London, UK; Centre for Clinical Brain Sciences, University of Edinburgh, Royal Edinburgh Hospital, UK

**Keywords:** Antidepressants, epidemiology, primary care, risk assessment, outcome studies

## Abstract

**Background:**

Antidepressants are one of the most widely prescribed drugs in the global north. However, little is known about the health consequences of long-term treatment.

**Aims:**

This study aimed to investigate the association between antidepressant use and adverse events.

**Method:**

The study cohort consisted of UK Biobank participants whose data was linked to primary care records (*N* = 222 121). We assessed the association between antidepressant use by drug class (selective serotonin reuptake inhibitors (SSRIs) and ‘other’) and four morbidity (diabetes, hypertension, coronary heart disease (CHD), cerebrovascular disease (CV)) and two mortality (cardiovascular disease (CVD) and all-cause) outcomes, using Cox's proportional hazards model at 5- and 10-year follow-up.

**Results:**

SSRI treatment was associated with decreased risk of diabetes at 5 years (hazard ratio 0.64, 95% CI 0.49–0.83) and 10 years (hazard ratio 0.68, 95% CI 0.53–0.87), and hypertension at 10 years (hazard ratio 0.77, 95% CI 0.66–0.89). At 10-year follow-up, SSRI treatment was associated with increased risks of CV (hazard ratio 1.34, 95% CI 1.02–1.77), CVD mortality (hazard ratio 1.87, 95% CI 1.38–2.53) and all-cause mortality (hazard ratio 1.73, 95% CI 1.48–2.03), and ‘other’ class treatment was associated with increased risk of CHD (hazard ratio 1.99, 95% CI 1.31–3.01), CVD (hazard ratio 1.86, 95% CI 1.10–3.15) and all-cause mortality (hazard ratio 2.20, 95% CI 1.71–2.84).

**Conclusions:**

Our findings indicate an association between long-term antidepressant usage and elevated risks of CHD, CVD mortality and all-cause mortality. Further research is needed to assess whether the observed associations are causal, and elucidate the underlying mechanisms.

Antidepressants are one of the most widely prescribed drugs. Seventy million prescriptions were dispensed in 2018, amounting to nearly a doubling of prescriptions in a decade.^1,2^ This striking rise in prescribing is attributed to long-term treatment rather than an increased incidence of depression,^3,4^ and these trends are not limited to the UK.^5–7^ To reduce the risk of relapse, until the 2022 revision of the guidelines, maintenance treatment was recommended of at least 6 months for patients who have recovered from depression, and at least 2 years for those identified at risk of recurrent depression.^8^ Some patients may also stay on treatment long term because of difficulties with discontinuation and infrequent monitoring.^9^ One Scottish study found that over half of patients on antidepressants had been taking them for more than 2 years, with a mean treatment duration of 5.5 years.^10^ However, little is known about the health consequences of long-term antidepressant treatment. There is *in vitro* evidence to suggest that some antidepressants have the potential to cause adverse cardiovascular and metabolic effects.^11–14^ Yet, most trials assessing the efficacy of antidepressants are poorly suited to examining adverse outcomes: they are often short term, are underpowered to look at most adverse outcomes, have methodological shortcomings^15,16^ and do not always report adverse effects, particularly serious ones.^15,17–21^ Depression is strongly associated with adverse risk profiles such as excess adiposity, smoking, poor diet and physical inactivity.^22,23^ These phenotypes and behaviours are established risk factors for a number of chronic conditions, including cardiovascular disease.^24^ Therefore, careful assessment of the long-term cardiometabolic effects of antidepressant treatment is critical.^25^ The main challenge for observational studies examining potential adverse outcomes of long-term antidepressant use is accounting for the excess cardiovascular risk associated with depression (confounding by indication). Studies have attempted to control for this confounding by limiting analyses to patients with a diagnosis of depression. However, there are considerable challenges relating to diagnostic validity, and not all primary care physicians give or record a diagnosis of depression even when it is recognised.^26^ Another approach is to identify and adjust for cardiometabolic risk factors that confound the association between depression and cardiometabolic outcomes. Meta-analyses of studies exploring the association between antidepressant use and a wide range of cardiometabolic outcomes reveal considerable heterogeneity between studies,^27–29^ and the evidence base remains weak.^25^ For example, a recent meta-analysis showed a 27% increased risk of diabetes with antidepressant use, but there was considerable variation in confounder adjustment within individual studies and none fully accounted for major risk factors and predictors for diabetes, including key markers of the metabolic syndrome.^27^

Given the multifactorial nature of depression and cardiometabolic disease,^30,31^ information on a wide range of prospectively measured confounders, including lifestyle, sociodemographic factors and baseline biomarkers for cardiometabolic disease, are needed to provide robust estimates of the risks associated with long-term antidepressant use. This requires richly phenotyped cohorts. One such cohort is UK Biobank, which is a large population-based cohort study (approximately 500 000 participants).^32^ This open-access resource has detailed information on socioeconomic status; demographics; anthropometric, behavioural and biochemical risk factors; disability and health status with linkages to routinely available national data-sets including primary care records and deaths. We used the UK Biobank data-set to examine the association between antidepressant use and four cardiometabolic morbidity outcomes (diabetes, hypertension, cerebrovascular disease (CV), coronary heart disease (CHD)) and two mortality outcomes (cardiovascular disease (CVD) mortality and all-cause mortality).

## Method

### Study cohort

UK Biobank recruited approximately 500 000 participants aged 40–69 years between 2006 and 2010. Our cohort was restricted to participants (*N* = 222 121) whose data had been linked to primary care records during the first phase of primary care data extraction (extracted in 2018, released 2019). Biobank participants who were registered with a general practitioner (GP) practice at least 12 months before study baseline and remained registered at study entry (Biobank entry date) were eligible for inclusion. Participants were excluded from this study if they had a prior recorded prescription for antidepressants (≤12 months before baseline); any prior recorded diagnosis for the outcome of interest; any prior recorded prescription for antipsychotics, lithium or antimanic drugs; or self-reported use of cardiometabolic drugs at baseline. We also excluded participants on antidepressant polytherapy. Participants entered our cohort at the Biobank baseline assessment date. Participants who did not have the event of interest within the follow-up period were censored at the earliest of date of death, date of leaving the GP practice or end of the follow-up period (either 5 or 10 years).

### Ethics and consent

UK Biobank has obtained ethics approval from the North West Multi-Centre Research Ethics Committee, which covers the UK (approval number: 11/NW/0382), and has obtained written informed consent from all participants.

### Exposure assessment

We extracted information on antidepressant use (antidepressant type, strength of medication, date of prescription and quantity prescribed) from linked primary care prescribing data focusing on ten of the most commonly prescribed antidepressants in England,^1^ with the exclusion of amitriptyline (often prescribed for pain or sleep problems in low doses) and dosulepin (not recommended in the UK national depression guidelines by the National Institute for Health and Care Excellence).^33^ Information on the dosing schedule was not available for extraction. The remaining eight antidepressants were categorised by drug class as selective serotonin reuptake inhibitors (SSRIs; citalopram, sertraline, fluoxetine, paroxetine) and ‘other’ antidepressants (mirtazapine, venlafaxine, duloxetine, trazodone). Antidepressant treatment was defined as a time-varying exposure (i.e. participants were classified as unexposed before their first antidepressant prescription, and subsequently classified as exposed at the date of the first antidepressant prescription). Antidepressant use was assessed in three ways: any antidepressant treatment, SSRI antidepressant treatment or other antidepressant treatment. To explore the dose–response relationship between antidepressant use and outcome, we calculated the number of defined daily doses (DDDs) by using values on the average maintenance dose assigned by the World Health Organization Collaborating Centre for Drug Statistics Methodology (www.whocc.no/atc_ddd_index). These were categorised as ≤0.5, >0.5 to 1.0 and >1.0. For example, for citalopram the DDD is 20 mg, 0.5 DDD is 10 mg and >1.0 DDD is anything >20 mg. In our study, DDD categories are referred to as low (≤0.5), intermediate (>0.5 to 1.0) and high (>1.0). For the purposes of calculating the DDD, we used the recorded product strength and estimated the prescribed daily dose from the total amount prescribed divided by the duration of treatment. The reference category was no antidepressant use and included unexposed time periods before starting treatment and unexposed time for those who did not receive an antidepressant prescription during follow-up.

### Outcome assessment

We selected four morbidity (diabetes, hypertension, CHD, CV) and two mortality (CVD and all-cause mortality) outcomes. Information on study outcomes were identified with relevant Read v2 and CTV3 codes (using the Quality and Outcomes Framework version 38; https://webarchive.nationalarchives.gov.uk/ukgwa/20220117164934/https://digital.nhs.uk/data-and-information/data-collections-and-data-sets/data-collections/quality-and-outcomes-framework-qof/quality-and-outcome-framework-qof-business-rules/quality-and-outcomes-framework-qof-business-rules-v-38-2017-2018-october-code-release) extracted from linked primary care records (diabetes, hypertension, CHD, CV) and ICD-10 codes extracted from death records (CVD, all-cause mortality). Outcomes were only included if they occurred after the date of entry into the cohort. We defined first incidence as the first recorded outcome during follow-up and with no prior recorded diagnosis for the outcome in primary care records and no self-reported diagnosis at baseline assessment. We initially planned to look at risk of outcome over 5 years; however, because of the small numbers of events, we extended our follow-up period to 10 years. We have included results from the 5-year follow-up to allow for comparison.

### Confounder assessment and selection

As highlighted earlier, depression – the main indication for antidepressants – is strongly associated with adverse risk profiles such as excess adiposity, smoking and physical inactivity. These are established risk factors for CVD and diabetes. To account for these shared risk factors, and given the multifactorial nature of cardiometabolic disease, we identified a wide range of personal, lifestyle, sociodemographic and biomarker covariates as potential confounders. These were age; gender; body mass index (BMI); waist/hip ratio; smoking and alcohol intake status; physical activity; parental history of outcome; biochemical and haematological biomarkers (apolipoproteins A and B, vitamin D, triglycerides, haemoglobin A1c); socioeconomic status (accommodation status, number of vehicles per household, employment status, benefits status, urban/rural status, education, household income) and self-reported long-term illness, disability or infirmity (as a generic measure of ‘ill health’). All confounders were assessed at baseline. Analyses were restricted to participants with non-missing information on confounders. Confounders for each outcome and follow-up period were selected with a stepwise approach through backward elimination, beginning with a model that included the main exposure of interest and all potential confounders. Except for age and gender (included in all models), confounders were retained where the Wald test was *P*≤0.05 and excluded if *P* > 0.05. Non-linear relationships between outcome and continuous confounders were considered by identifying, at each iterative step of the stepwise process, the best-fitting fractional polynomial terms. Details of the selected confounders for each model are shown in Supplementary Appendix 1 available at https://doi.org/10.1192/bjo.2022.563.

### Sensitivity analysis

We did not have sufficient numbers to compare the effects of short-term and long-term antidepressant usage. However, we carried out a sensitivity analysis to exclude short-term usage (<90 days) and get a better sense of long-term chronic effects, i.e. related to metabolic dysfunction.

### Statistical analysis

All analyses were conducted in Stata version 16 for Windows. The association between antidepressant treatment and each outcome (diabetes, hypertension, CV, CHD, CVD mortality and all-cause mortality) was quantified using Cox's proportional hazards model, with study duration as the underlying timescale. Antidepressant treatment was treated as a time-varying exposure. Hazard ratios and 95% confidence intervals were estimated for each antidepressant treatment category (any, SSRI, other) and outcome. Results are first provided from a model adjusting for baseline age and gender, and then from the fully adjusted model with the confounders selected by the multivariable selection procedure described above. We also estimated hazard ratio and 95% confidence interval by DDD category for the 10-year follow-up (insufficient number of events at 5-year follow-up). The proportional hazards assumption was assessed by means of the scaled Schoenfeld residuals, which were used to test the proportionality over time for each covariate in the final model being fitted. If there was evidence at the 5% level of a violation of the proportional hazards assumption for any covariate, the final model was refitted to include a time-varying coefficient (i.e. an interaction term between that particular covariate and time). Kaplan–Meier curves for each of the outcomes are shown in Supplementary Appendix 2.

## Results

**Fig. 1:**
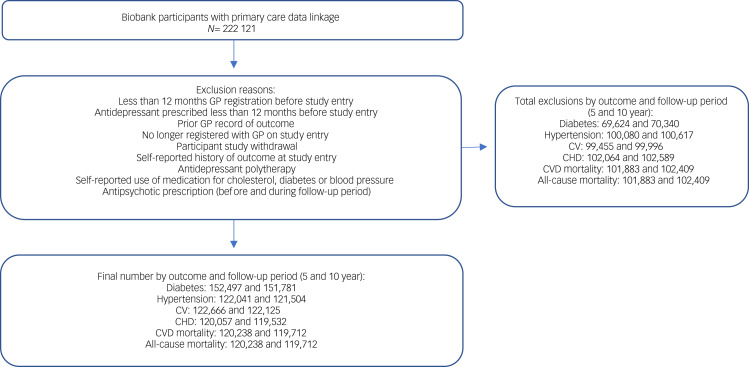
Cohort selection. CHD, coronary heart disease; CV, cerebrovascular disease; CVD, cardiovascular disease; GP, general practitioner.

Biobank participants who had linked primary care data were similar to participants without such data in terms of key sociodemographic and clinical characteristics (age, gender, ethnicity, socioeconomic status, BMI, long-term illness) (Supplementary Appendix 3). The number of participants in the final study cohort varied by outcome (Fig. 1). Baseline participant characteristics are presented for each outcome cohort in Table 1. On average (median) participants were aged 56–57 years, around half of the participants or just over were female, and 96% were of White ethnicity.

**Table 1:** Participant baseline characteristics at 5-year follow-up

Characteristic	Diabetes (*n* = 152 497)	Hypertension (*n* = 122 041)	CV (*n* = 122 666)	CHD (*n* = 120 057)	CVD mortality (*n* = 120 238)	All-cause mortality (*n* = 120 238)
Age (years), median (IQR)	57 (49–63)	56 (48–62)	56 (49–62)	56 (48–62)	56 (48–62)	56 (48–62)
Gender, *n* (%)						
Male	76 948 (50.5)	53 219 (43.6)	53 587 (43.7)	51 670 (43.0)	51 813 (43.1)	51 813 (43.1)
Female	75 549 (49.5)	68 822 (56.4)	69 079 (56.3)	68 387 (57.0)	68 425 (56.9)	68 425 (56.9)
Ethnic group, *n* (%)						
White	146 057 (96.2)	116 665 (96.0)	117 206 (96.0)	115 227 (96.3)	115 399 (96.3)	115 399 (96.3)
Ethnic minority	5755 (3.8)	4811 (4.0)	4861 (4.0)	4494 (3.7)	4501 (3.7)	4501 (3.7)
Apolipoprotein A (g/L), mean (s.d.)	1.5 (0.3)	1.5 (0.3)	1.5 (0.3)	1.5 (0.3)	1.5 (0.3)	1.5 (0.3)
Apolipoprotein B (g/L), mean (s.d.)	1.0 (0.2)	1.0 (0.2)	1.0 (0.2)	1.0 (0.3)	1.1 (0.2)	1.1 (0.2)
Haemoglobin A1c (mmol/mol), median (IQR)	34.9 (32.5–37.2)	34.7 (32.4–37.1)	34.7 (32.4–37.1)	34.6 (32.3–37.0)	34.6 (32.3–37.0)	34.6 (32.3–37.0)
BMI (kg/m^2^), median (IQR)	26.5 (24.0–29.5)	26.1 (23.7–28.9)	26.1 (23.7–28.9)	26.1 (23.7–28.9)	26.0 (23.7–28.9)	26.0 (23.7–28.9)
WHR, median (IQR)	0.87 (0.80–0.94)	0.86 (0.79–0.92)	0.86 (0.79–0.92)	0.85 (0.79–0.92)	0.86 (0.79–0.92)	0.86 (0.79–0.92)
Triglycerides (mmol/L), median (IQR)	1.46 (1.03–2.12)	1.47 (1.00–2.04)	1.41 (1.00–2.04)	1.4 (1.0–2.0)	1.40 (1.0–2.0)	1.40 (1.0–2.0)
Vitamin D (nmol/L), median (IQR)	46.9 (32.5–62.4)	47.0 (32.5–62.4)	46.9 (32.5–62.4)	47.1 (32.6–62.5)	47.1 (32.6–62.5)	47.1 (32.6–62.5)
Long-standing illness, *n* (%)						
No	107 350 (72.1)	90 123 (75.5)	90 458 (75.4)	89 751 (76.3)	89 843 (76.2)	89 843 (76.2)
Yes	41 552 (27.9	29 277 (24.5)	29 515 (24.6)	27 955 (23.7)	28 040 (23.8)	28 040 (23.8)
Ever smoked, *n* (%)						
No	61 895 (40.8)	51 453 (42.4)	51 699 (42.4)	50 794 (42.4)	50 855 (42.4)	50 855 (42.4)
Yes	89 902 (59.2)	70 009 (57.6)	70 351 (57.6)	68 913 (57.6)	69 033 (57.6)	69 033 (57.6)
Alcohol intake, *n* (%)						
Never	10 348 (6.8)	8439 (6.9)	8507 (6.9)	8151 (6.8)	8168 (6.8)	8168 (6.8)
Special occasions only	15 102 (9.9)	12 438 (10.2)	12 517 (10.2)	12 172 (10.1)	12 191 (10.1)	12 191 (10.1)
1–3 times per month	16 713 (11.0)	13 984 (11.5)	14 044 (11.5)	13 777 (11.5)	13 797 (11.5)	13 797 (11.5)
Once or twice a week	41 117 (27.0)	33 446 (27.5)	33 616 (27.5)	33 026 (27.5)	33 069 (27.5)	33 069 (27.5)
3–4 times a week	37 419 (24.6)	29 725 (24.4)	29 842 (24.4)	29 409 (24.5)	29 450 (24.5)	29 450 (24.5)
Daily	31 456 (20.7)	23 718 (19.5)	23 818 (19.5)	23 455 (19.5)	23 495 (10.6)	23 495 (10.6)
Number of days/week of moderate physical activity, *n* (%)						
0	17 824 (12.3)	13 830 (11.9)	13 914 (11.9)	13 540 (11.8)	13 563 (11.8)	13 563 (11.8)
1	11 635 (8.0)	9408 (8.1)	9440 (8.0)	9251 (8.1)	9261 (8.1)	9261 (8.1)
2–3	43 323 (29.8)	35 167 (30.2)	35 312 (30.2)	34 718 (30.2)	34 762 (30.2)	34 762 (30.2)
4–5	36 946 (25.4)	29 515 (25.4)	29 658 (25.4)	29 171 (25.4)	29 214 (25.4)	29 214 (25.4)
6–7	35 498 (24.4)	28 450 (24.4)	28 599 (24.5)	28 092 (24.5)	28 141 (24.5)	28 141 (24.5)
Parental history,^a^ *n* (%)						
No	131 802 (86.4)	86 802 (71.1)	110 316 (89.9)	98 445 (82.0)	47 156 (39.2)	47 156 (39.2)
Yes	20 695 (13.6)	35 239 (28.9)	12 350 (10.1)	21 612 (18.0)	73 082 (60.8)	73 082 (60.8)
Type of accommodation, *n* (%)						
House	138 745 (91.4)	111 365 (91.6)	111 897 (91.6)	109 821 (91.7)	109 977 (91.7)	109 977 (91.7)
Flat/apartment	12 765 (8.4)	9927 (8.2)	9987 (8.2)	9695 (8.1)	9719 (8.1)	9719 (8.1)
Other	359 (0.2)	240 (0.2)	243 (0.2)	234 (0.2)	234 (0.2)	234 (0.2)
Household vehicles, *n* (%)						
None	10 601 (7.0)	8006 (6.6)	8067 (6.6)	7792 (6.5)	7807 (6.5)	7807 (6.5)
One	62 830 (41.5)	49 416 (40.8)	49 679 (40.8)	48 689 (40.8)	48 771 (40.8)	48 771 (40.8)
Two	60 717 (40.1)	49 560 (40.9)	49 777 (40.9)	48 944 (41.0)	49 010 (50.0)	49 010 (50.0)
Three or more	17 282 (11.4)	14 210 (11.7)	14 257 (11.7)	14 008 (11.7)	14 024 (11.7)	14 024 (11.7)
Employment status, *n* (%)						
Paid	92 616 (61.3)	77 652 (64.3)	77 914 (64.2)	76 683 (64.4)	76 762 (64.4)	76 762 (64.4)
Retired	47 225 (31.3)	34 478 (28.5)	34 763 (28.6)	33 953 (28.5)	34 048 (28.5)	34 048 (28.5)
Unpaid role/student	4998 (3.3)	4481 (3.7)	4492 (3.7)	4411 (3.7)	4413 (3.7)	4413 (3.7)
Unable to work	3889 (2.6)	2477 (2.0)	2503 (2.1)	2376 (2.0)	2379 (2.0)	2379 (2.0)
Unemployed	2266 (1.5)	1732 (1.4)	1742 (1.4)	1660 (1.4)	1661 (1.4)	1661 (1.4)
Educational qualifications, *n* (%)						
School level	40 258 (31.9)	33 019 (32.0)	33 146 (40.0)	25 863 (25.4)	25 885 (25.4)	25 885 (25.4)
NVQ/HND or equivalent	10 650 (8.4)	7746 (7.5)	7788 (7.5)	7589 (7.4)	7601 (7.4)	7601 (7.4)
A level or equivalent	16 846 (13.3)	13 912 (13.5)	13 969 (13.5)	20 460 (20.1)	20 486 (20.1)	20 486 (20.1)
University/professional	58 631 (46.4)	48 591 (47.0)	48 781 (47.0)	47 990 (47.1)	48 055 (47.1)	48 055 (47.1)
Disability allowance, *n* (%)						
No benefits	144 265 (95.5)	116 671 (96.5)	117 185 (96.5)	115 148 (96.6)	115 307 (96.6)	115 307 (96.6)
Disability benefits	6752 (4.5)	4247 (3.5)	4306 (3.5)	4089 (3.4)	4108 (3.4)	4108 (3.4)
Urban/rural home status, *n* (%)						
Rural/small town	23 973 (15.9)	19 320 (16.0)	19 391 (16.0)	19 089 (16.0)	19 114 (16.0)	19 114 (16.0)
Urban	127 210 (84.1)	101 642 (84.0)	102 189 (84.0)	99 904 (84.0)	100 060 (84.0)	100 060 (84.0)
Household income (£), *n* (%)						
<18 000	27 504 (20.8)	20 304 (19.2)	20 449 (19.3)	19 866 (19.1)	19 904 (19.1)	19 904 (19.1)
18 000–30 999	33 938 (25.7)	26 450 (25.1)	26 582 (25.1)	26 045 (25.0)	26 088 (25.0)	26 088 (25.0)
31 000–51 999	36 104 (27.4)	29 593 (28.0)	29 698 (28.0)	29 268 (28.1)	29 300 (28.1)	29 300 (28.1)
52 000–100 000	27 653 (20.9)	23 391 (22.2)	23 465 (22.1)	23 156 (22.2)	23 179 (22.2)	23 179 (22.2)
>100 000	6825 (5.2)	5855 (5.5)	5873 (5.5)	5781 (5.6)	5792 (5.6)	5792 (5.6)

CV, cerebrovascular disease; CHD, coronary heart disease; CVD, cardiovascular disease; IQR, interquartile range; BMI, body mass index; WHR, waist/hip ratio; NVQ/HND, National Vocational Qualification/Higher National Diploma.

a. Parental history of outcome, for mortality outcomes this is parental history of cardiometabolic and vascular disease.

On average, 8% of participants in each cohort had been prescribed an antidepressant by the 5-year follow-up and 6% by the 10-year follow-up (Supplementary Appendix 4). SSRIs were the most commonly prescribed antidepressant class (80–82%), and citalopram was the most commonly prescribed SSRI (46–47%). Mirtazapine was the most frequently prescribed antidepressant in the ‘other’ category (44–46%) (Supplementary Appendix 4).

The number of events, person-years of follow-up and hazard ratios (95% confidence intervals) for the six outcomes by antidepressant class are presented in Tables 2 and 3 for the 5- and 10-year follow-ups, respectively.

**Table 2:** Hazard ratios by outcome and antidepressant class at 5-year follow-up

					Age and gender adjusted		Fully adjusted	
Antidepressant class	Participants	Observations	Events	Person-years	Hazard ratio	95% CI	Hazard ratio	95% CI
Diabetes	114 076	124 239	961	545 735				
No antidepressant			872	495 551	1.00			
Any antidepressant			89	50 185	1.28	(1.03–1.59)	0.83	(0.66–1.04)
SSRIs			62	46 557	0.91	(0.72–1.14)	0.64	(0.49–0.83)
Other antidepressant			38	11 503	2.03	(1.47–2.81)	1.13	(0.81–1.57)
Hypertension	107 316	117 025	2621	508 852				
No antidepressant			2411	460 887	1.00		1.00	
Any antidepressant			210	47 965	1.02	(0.89–1.18)	0.93	(0.81–1.08)
SSRIs			161	38 986	1.02	(0.89–1.18)	0.91	(0.77–1.07)
Other antidepressant			49	8991	1.15	(0.87–1.53)	1.02	(0.77–1.36)
CV	121 190	132 313	337	581 598				
No antidepressant			306	526 413	1.00		1.00	
Any antidepressant			31	55 186	1.17	(0.81–1.70)	1.04	(0.72–1.52)
SSRIs			25	43 784	1.24	(0.86–1.79)	1.12	(0.74–1.69)
Other antidepressant			7	10 619	1.18	(0.56–2.49)	1.15	(0.54–2.43)
CHD	80 964	87 972	545	388 540				
No antidepressant			492	353 972	1.00		1.00	
Any antidepressant			53	34 568	1.63	(1.22–2.17)	1.47	(1.10–1.95)
SSRIs			41	28 448	1.55	(1.15–2.08)	1.44	(1.04–1.99)
Other antidepressant			13	6177	1.78	(1.03–3.09)	1.59	(0.91–2.77)
CVD mortality	113 800	124 084	150	546 926				
No antidepressant			135	495 849	1.00		1.00	
Any antidepressant			15	51 077	1.53	(0.89–2.61)	1.21	(0.70–2.10)
SSRIs			11	41 490	1.24	(0.68–2.25)	1.16	(0.62–2.18)
Other antidepressant			4	10 220	1.50	(0.56–4.05)	1.14	(0.42–3.11)
All-cause mortality	101 609	110 788	746	488 286				
No antidepressant			649	442 834	1.00		1.00	
Any antidepressant			97	45 452	1.79	(1.45–2.23)	1.37	(1.10–1.70)
SSRIs			76	36 649	1.44	(1.14–1.83)	1.41	(1.11–1.80)
Other antidepressant			27	9686	1.89	(1.29–2.78)	1.30	(0.88–1.92)

SSRI, selective serotonin reuptake inhibitor; CV, cerebrovascular disease; CHD, coronary heart disease; CVD, cardiovascular disease.

**Table 3:** Hazard ratios by outcome and antidepressant class at 10-year follow-up

					Age and gender adjusted		Fully adjusted	
Antidepressant class	Participants	Observations	Events	Person-years	Hazard ratio	95% CI	Hazard ratio	95% CI
Diabetes	98 338	109 681	1456	890 085				
No antidepressant			1352	817 492	1.00			
Any antidepressant			104	72 592	1.12	(0.92–1.37)	0.77	(0.63–0.94)
SSRIs			72	59 606	1.13	(0.94–1.36)	0.68	(0.53–0.87)
Other antidepressant			47	15 814	2.01	(1.50–2.68)	1.17	(0.87–1.57)
Hypertension	92 524	103 416	3777	825 174				
No antidepressant			3545	755 910	1.00		1.00	
Any antidepressant			232	69 263	0.86	(0.75–0.98)	0.80	(0.70–0.91)
SSRIs			182	57 514	0.83	(0.73–0.94)	0.77	(0.66–0.89)
Other antidepressant			51	11 835	1.19	(0.97–1.46)	0.96	(0.73–1.26)
CV	105 536	118 030	693	962 201				
No antidepressant			625	882 022	1.00		1.00	
Any antidepressant			68	80 179	1.42	(1.10–1.83)	1.26	(0.97–1.62)
SSRIs			57	66 283	1.44	(1.13–1.84)	1.34	(1.02–1.77)
Other antidepressant			11	14 035	1.12	(0.62–2.04)	0.97	(0.54–1.77)
CHD	79 938	89 029	1015	728 001				
No antidepressant			934	669 588	1.00		1.00	
Any antidepressant			81	58 413	1.49	(1.18–1.87)	1.32	(1.04–1.66)
SSRIs			56	76 620	1.38	(1.10–1.73)	1.15	(0.87–1.51)
Other antidepressant			23	9320	2.25	(1.48–3.40)	1.99	(1.31–3.01)
CVD mortality	103 567	115 732	454	947 147				
No antidepressant			389	868 874	1.00		1.00	
Any antidepressant			65	78 273	2.54	(1.94–3.32)	1.89	(1.44–2.49)
SSRIs			50	64 864	1.92	(1.43–2.57)	1.87	(1.38–2.53)
Other antidepressant			15	13 409	2.71	(1.62–4.55)	1.86	(1.10–3.15)
All-cause mortality	81 279	90 508	1752	745 059				
No antidepressant			1511	685 708	1.00		1.00	
Any antidepressant			241	59 352	2.23	(1.94–2.56)	1.86	(1.61–2.14)
SSRIs			181	49 651	1.68	(1.44–1.96)	1.73	(1.48–2.03)
Other antidepressant			63	10 127	2.82	(2.19–3.63)	2.20	(1.71–2.84)

SSRI, selective serotonin reuptake inhibitor; CV, cerebrovascular disease; CHD, coronary heart disease; CVD, cardiovascular disease.

At 5 years (Table 2), in models that adjusted for age and gender, any antidepressant use was associated with an increased risk of diabetes (hazard ratio 1.28, 95% CI 1.03–1.59), CHD (hazard ratio 1.63, 95% CI 1.22–2.17) and all-cause mortality (hazard ratio 1.79, 95% CI 1.45–2.23), with only weak evidence of an increased risk of CVD mortality (hazard ratio 1.53, 95% CI 0.89–2.61), with the confidence interval for the latter including the null. For CHD and all-cause mortality, findings were attenuated after further adjustment for confounders (CHD: hazard ratio 1.47, 95% CI 1.10–1.95; all-cause mortality: hazard ratio 1.37, 95% CI 1.10–1.70), and were no longer evident in the fully adjusted models for diabetes (hazard ratio 0.83, 95% CI 0.66–1.04) or CVD mortality (hazard ratio 1.21, 95% CI 0.70–2.10). Looking at antidepressants by class (SSRIs and ‘other’) did not change the overall pattern of results for CHD and all-cause mortality. ‘Other’ antidepressants were associated with an increased risk of diabetes in the model adjusting for age and gender (hazard ratio 2.03, 95% CI 1.47–2.81), but this was no longer evident in the fully adjusted model (hazard ratio 1.13, 95% CI 0.81–1.57). However, SSRIs were weakly associated with a reduced risk of diabetes in the model adjusted for age and gender, which became stronger following full adjustment (hazard ratio 0.64, 95% CI 0.49–0.83). There was no clear evidence of any association between antidepressant use and either hypertension or CV, although the number of CV outcomes was small (*n* = 31).

Antidepressant treatment was similarly associated with an increased risk of CHD and all-cause mortality at 10 years (Table 3), and these effects were only slightly attenuated in the fully adjusted model.

Antidepressants in the ‘other’ class were associated with a higher risk of these outcomes. There was a weak association between any antidepressant use and incident diabetes (hazard ratio 1.12, 95% CI 0.92–1.37), but the direction of this effect was reversed following adjustment for all confounders (hazard ratio 0.77, 95% CI 0.63–0.94). Similar findings were observed for SSRIs, but ‘other’ antidepressants were associated with a small increased risk of diabetes, although the confidence interval included the null (hazard ratio 1.17, 95% CI 0.87–1.57). There was also evidence that any antidepressant use was associated with an increased risk of both CV and CVD mortality at 10 years, which attenuated only slightly following full adjustment (CV: hazard ratio 1.26, 95% CI 0.97–1.62; CVD mortality: hazard ratio 1.89, 95% CI 1.44–2.49). Looking at antidepressant class, this effect was only observed for SSRIs and CV (fully adjusted hazard ratio 1.34, 95% CI 1.02–1.77), but was seen with both antidepressant groups for CVD mortality. In contrast, any antidepressant use was associated with a reduction in incident hypertension (fully adjusted hazard ratio 0.80, 95% CI 0.70–0.91), as was SSRI use (hazard ratio 0.77, 95% CI 0.66–0.89).

There was some evidence of a dose–response effect (Table 4) for all-cause mortality, with higher doses associated with an increased risk of this outcome. This was reflected in the analysis of the two antidepressant classes. A similar pattern was evident for CV and CHD, but the results were subject to considerable uncertainty. There was no clear evidence of a dose-response effect for the other outcomes.

**Table 4:** Defined daily dose hazard ratios by outcome and antidepressant class at 10-year follow-up

	Any antidepressant				SSRI class				Other class			
DDD category	Events	Person-years	Hazard ratio	95% CI	Events	Person-years	Hazard ratio	95% CI	Events	Person-years	Hazard ratio	95% CI
Diabetes												
No antidepressant	1352	817 492	1.00		1352	817 492	1.00		1681	979 614	1.00	
≤0.5	21	40 984	0.34	(0.22–0.53)	15	33 374	0.32	(0.19–0.54)	10	9285	0.54	(0.29–1.00)
>0.5 to 1.0	7	15 306	0.23	(0.11–0.49)	4	13 140	0.15	(0.06–0.41)	4	2683	0.65	(0.24–1.74)
>1.0	56	15 924	0.65	(0.33–1.31)	40	12 945	1.45	(1.05–1.99)	23	3760	0.31	(0.09–1.04)
Hypertension												
No antidepressant	3563	759 428	1.00		3563	759 428	1.00		3563	759 428	1.00	
≤0.5	80	40 415	0.51	(0.40–0.63)	62	33 114	0.49	(0.38–0.63)	18	7301	0.58	(0.36–0.92)
>0.5 to 1.0	29	14 487	0.13	(0.04–0.41)	21	12 605	0.40	(0.26–0.61)	8	1883	0.90	(0.45–1.80)
>1.0	88	14 458	0.55	(0.33–0.93)	69	11 893	0.55	(0.30–0.99)	19	2565	1.49	(0.95–2.35)
CV												
No antidepressant	625	882 022	1.00		625	882 022	1.00		630	885 445	1.00	
≤0.5	29	882 022	0.95	(0.66–1.39)	25	38 023	1.08	(0.72–1.62)	4	8644	0.56	(0.21–1.51)
>0.5 to 1.0	12	46 576	1.09	(0.61–1.93)	9	14 591	0.98	(0.51–1.89)	3	2255	1.74	(0.56–5.43)
>1.0	18	16 828	1.45	(0.90–2.32)	15	13 377	1.51	(0.90–2.52)	3	3047	1.24	(0.40–3.87)
CHD												
No antidepressant	938	673 779	1.00		938	673 779	1.00		887	638 727	1.00	
≤0.5	31	34 372	0.90	(0.63–1.29)	20	28 340	0.75	(0.48–1.16)	11	5711	1.53	(0.84–2.79)
>0.5 to 1.0	16	12 335	1.24	(0.75–2.03)	11	10 716	1.01	(0.56–1.84)	4	1516	2.03	(0.76–5.45)
>1.0	24	11 819	1.73	(1.15–2.60)	19	9727	1.71	(1.08–2.71)	5	2000	1.92	(0.79–4.65)
CVD mortality												
No antidepressant	426	903 678	1.00		392	872 950	1.00		426	903 678	1.00	
≤0.5	37	47 545	1.73	(1.23–2.44)	25	37 227	1.76	(1.17–2.65)	9	8710	1.56	(0.80–3.03)
>0.5 to 1.0	12	17 529	1.56	(0.87–2.78)	10	14 393	1.73	(0.92–3.26)	2	2278	1.46	(0.36–5.87)
>1.0	15	17 300	1.55	(0.92–2.63)	8	13 370	1.21	(0.60–2.46)	6	3143	3.06	(1.35–6.93)
All-cause mortality												
No antidepressant	1364	638 409	1.00		1511	685 708	1.00		1566	705 811	1.00	
≤0.5	93	32 768	1.38	(1.12–1.71)	81	28 818	1.40	(1.12–1.76)	29	6260	1.60	(1.10–2.31)
>0.5 to 1.0	55	11 775	2.33	(1.77–3.06)	40	10 873	1.78	(1.30–2.45)	16	1637	3.34	(2.04–5.49)
>1.0	55	11 303	2.12	(1.61–2.79)	51	9752	2.15	(1.62–2.86)	15	2155	2.70	(1.61–4.50)

DDD, defined daily dose; SSRI, selective serotonin reuptake inhibitor; CV, cerebrovascular disease; CHD, coronary heart disease; CVD, cardiovascular disease.

The results of the sensitivity analysis removing individuals with short periods of antidepressant use did not have a marked effect on the associations of interest (Supplementary Appendix 5).

## Discussion

### Brief summary of the main findings

This population-based cohort study investigated whether commonly prescribed antidepressants were associated with a risk of developing diabetes, hypertension, CV, CHD and mortality (CVD and all-cause). Our study found that long-term antidepressant use was associated with an increased risk of CHD, CVD and all-cause mortality. These issues appear to be more problematic for antidepressants other than SSRIs (mirtazapine, venlafaxine, duloxetine, trazodone), with the use of such drugs associated with a two-fold increased risk of CHD, CVD and all-cause mortality at 10 years. There was also some evidence that antidepressants, and particularly SSRIs, were associated with a reduced risk of developing hypertension and diabetes. The findings were particularly evident after 10 years of follow-up, where we had larger numbers of events.

Comparison of hazard ratios before and after adjustment for a large number of confounders suggested that the associations between antidepressant use and increased risk of diabetes are confounded by adverse clinical phenotypes commonly associated with depression. For diabetes, this confounding appears to be driven by key metabolic risk markers and factors for this condition, mainly haemoglobin A1C and BMI.^34,35^ This is less apparent for hypertension, where there was little difference in estimates before and after adjustment for all confounders.

### Comparison with existing studies

Previous meta-analyses have highlighted the challenges of comparing work in this field because of significant heterogeneity in study design and methods, including measurement of exposures, outcomes and adjustment for confounders.^25^ A systematic review and meta-analysis of randomised trials found that SSRIs were associated with an improvement in glycemia, which was not moderated by depression status, diabetes status or change in weight across studies.^36^ This work is consistent with our findings of a lower risk of diabetes with SSRI treatment. In contrast, a meta-analysis^27^ and pharmaco-vigilance study^37^ both found an association between treatment with antidepressants and increased rates of diabetes. The conflicting findings between these studies and our work could be explained by differences in the adjustment for confounders. For example, in the meta-analysis, none of the included studies adjusted for haemoglobin A1C and less than half adjusted for BMI.^27^ The lack of adjustment of key risk factors associated with depression – the main indication for treatment – and outcomes suggests a lack of sufficient control for confounding by indication in previous work. This applies to previous work related to all of our studied outcomes.

There is some evidence that depression is associated with lower blood pressure,^38,39^ although this is contradicted by a meta-analysis of prospective cohort studies that concluded depression ‘is probably a risk factor for hypertension’.^40^ Licht et al also found that antidepressant use increased the risk of developing hypertension, whereas the other studies mentioned did not adjust for antidepressant use.^38,39^ Our study is not in line with the findings of Licht et al regarding the effect of antidepressant treatment. We found a reduction in the risk of developing hypertension for the ‘any’ antidepressants and ‘SSRI’ categories, although it was less convincing for the category of ‘other’ antidepressants.

Coupland et al^41,42^ described an increased risk of CV with antidepressants in those with incident prescriptions who were aged >65 years. However, these studies were based on primary care data and adjusted for a limited range of confounders. A meta-analysis also found that SSRIs were associated with an increased risk of CV (relative risk 1.24, 95% CI 1.15–1.34).^28^ The results of this meta-analysis should be treated with caution because the estimates are characterised by a high between-study heterogeneity. Moreover, it was not possible to distinguish between the effects of antidepressants and depression itself. Our fully adjusted model at 10-year follow-up, although showing a trend toward increased risk, also includes the possibility of no association (hazard ratio 1.26, 95% CI 0.97–1.62) for all antidepressants. However, there was some evidence of an increase in risk for those on SSRIs (hazard ratio 1.34, 95% CI 1.02–1.77). Although this may reflect a genuine risk from this class of drug, it may also be because SSRIs are widely perceived as safer than other antidepressants, and are therefore more likely to be prescribed to those who are already at risk.

Our finding of an increase in the risk of developing CHD and of CVD mortality is broadly in line with published work.^43^ However, there are some differences. Oh et al^44^ highlighted the risk of tricyclic antidepressants in CHD and found that SSRIs did not increase risk, although most of their evidence came from case–control designs and studies that were scored as low quality. Our findings are more nuanced. We found an increase in the risk of CHD and CVD mortality in the 10-year fully adjusted model, except for CHD in those taking SSRIs, where confidence intervals included the null. We did not replicate Coupland et al's finding of a reduced risk of myocardial infarction in a younger cohort.^41^

Our finding of an increase in all-cause mortality at 10-year follow-up is supported by Almeida et al.^45^ They described a risk that increases with the severity of depression. In their study, those who were currently well and taking antidepressants were at lower risk than those who were depressed, irrespective of whether they were taking antidepressants. This suggests that other factors related to depression (for example, suicidality) may be more important contributors to all-cause mortality than antidepressants. Our study design does not allow us to determine this.

### Strengths and limitations

The major strength of this study is the linkage of a richly phenotyped national prospective cohort study to primary care records. This has enabled us to examine multiple cardiometabolic outcomes, and thus obtain a more complete picture on the potential long-term effects of taking antidepressant medication. Importantly, the use of the linked data from Biobank has enabled adjustment for a wide range of prospectively measured confounders – both clinical and socioeconomic – in our analytical models. In addition, this data linkage ensured the availability of high-quality data in terms of measures of exposure and outcomes. We have therefore been able to overcome the limitations of previous work that have relied solely on prospective cohorts (with poorly measured exposures/outcomes) and those that utilised data from primary care records (with limited information on confounders).

Limitations include the time lag between measurement of confounders and outcomes (up to 5 and 10 years). We did not have enough events to carry out a sensitivity analysis at 1-year follow-up to allow us to assess the impact of this. Furthermore, although we adjusted for a wide range of potential confounders, we cannot rule out the possibility of residual confounding. The low number of events meant that we could not compare outcomes by individual antidepressants. However, we were able to explore findings by antidepressant class. Like previous work, we did not have information on the severity of depression at the time of prescription and outcome. This information is not included routinely in primary care records. No information on dosing schedule was available. However, we calculated DDDs by using WHO data to explore dose–response effects. Patients may not have taken their antidepressant medication as prescribed, and therefore it is possible that there may be some misclassification of the antidepressant exposure. Type 1 errors might be inflated from testing of multiple outcomes, and significant findings should be interpreted with caution.

Finally, only 44% of the Biobank had linked primary care data available at the time of our analyses and this could have introduced bias. However, comparison of baseline characteristics between those with and without linked primary care data suggested few differences. Our study cohort is mostly of White British ethnic origin and our findings require replication in more ethnically diverse cohorts, particularly given ethnic differences in cardiometabolic risk and disease.

### Clinical implications

Antidepressants, and especially SSRIs, may have a good safety profile in the short term, but are associated with adverse outcomes in the long term. This is important because most of the substantial increase in prescribing in the past 20 or more years is in long-term repeat prescribing. Although we cannot establish causality, we have described concerning associations with increases in CHD, CVD and all-cause mortality that are broadly in line with earlier findings, but have been undertaken in a cohort that has had detailed prospectively recorded information, enabling us to adjust for important confounders. The increase in all-cause mortality is also worrying, although, as we note above, other factors related to depression (for example suicidality) may be more important contributors to all-cause mortality than antidepressants. Some of our findings are less concerning. We found some evidence that, once other clinical and socioeconomic factors are adjusted for, antidepressants – and particularly SSRIs – may reduce the risk of developing hypertension and diabetes. This is intriguing and, if it is supported, suggests directions for research into the mechanisms involved in the association between antidepressants and CHD and CVD mortality. Since this is an observational study, our findings do not imply causality, and highlight the importance of further work to investigate and elucidate potential mechanisms. In the meantime, the message for clinicians is that prescribing of antidepressants in the long term may not be harm-free, and it is particularly important to review the cardiovascular health of patients on antidepressants more proactively and have discussions around stopping treatment for those on long-term treatment, particularly those with CVD.

## Data Availability

The data was created under UK Biobank application number 46704 and is available directly from UK Biobank.

## Supplementary material

For supplementary material accompanying this paper visit http://doi.org/10.1192/bjo.2022.563. click here to view supplementary material
