# Supplementary material for: Antidepressant use and risk of adverse outcomes: population-based cohort study
Source: BJPsych Open. 2022 Sep 13;8(5):e164. doi: 10.1192/bjo.2022.563 (PMC9534882; doi:10.1192/bjo.2022.563)
Supplement: Supplementary file 1 [file bjosup.zip › S2056472422005634sup001.docx]

**Supplementary**

Appendix 1a: List of confounders included in the models looking at the exposure of any antidepressant treatment

Footnote: ^$^non-linear term *p<0.05, **p<0.01, ***p<0.001

Appendix 1b: List of confounders included in the models looking at the exposure of SSRI treatment

Footnote: ^$^non-linear term *p<0.05, **p<0.01, ***p<0.001

Appendix 1c: List of confounders included in the models looking at the exposure of ‘Other’ class of antidepressants

Appendix 2a: Kaplan Meier curves at 5-year follow up

Appendix 2b: Kaplan Meier curves at 10-year follow up

Appendix 3: Biobank participant characteristics by primary care data linkage status

Appendix 4: Antidepressant prescriptions by cohort

Footnote: ^$^non-linear term *p<0.05, **p<0.01, ***p<0.001

Appendix 5: Sensitivity analysis excluding short term antidepressant use (<90 days)
