# Supplementary material for: Antidepressant use and risk of adverse outcomes: population-based cohort study
Source: BJPsych Open. 2022 Sep 13;8(5):e164. doi: 10.1192/bjo.2022.563 (PMC9534882; doi:10.1192/bjo.2022.563)
Supplement: Supplementary file 1 [file bjosup.zip › S2056472422005634sup002.docx]

Appendix 1: Biobank participant characteristics by primary care data linkage status

|  | Linked  (n=222,121) | Unlinked  (n=280,403) |
| --- | --- | --- |
| Age (years): median [IQR] | 58 [50-63] | 58 [50-63] |
| Male: n (%) | 100,149 (45.1%) | 128,980 (46.0%) |
| White: n (%) | 210,733 (95.3%) | 261,981 (94.0%) |
| University/professional education: n (%) | 81,917 (45.7%) | 105,057 (46.1%) |
| Urban location: n (%) | 186,597 (84.9%) | 242,203 (87.2%) |
| Disability allowance: n (%) | 13,925 (6.3%) | 16,089 (5.8%) |
| Median BMI (kg/m^2^): median [IQR] | 26.8 [24.2-30.0] | 26.6 [24.1-29.8] |
| Long term illness: n (%) | 72,599 (33.6%) | 87,307 (32.0%) |

Appendix 2: Antidepressant prescriptions by outcome

|  | Diabetes | Hypertension | CVA | CHD | CVD mortality | ACM |
| --- | --- | --- | --- | --- | --- | --- |
| 5-year | | | | | | |
| Antidepressant  N (%) | 11,978 (7.9) | 9,582 (7.9) | 9,709 (7.9) | 9,406 (7.8) | 9,559 (7.9) | 9,546 (7.9) |
| SSRI  N (% of AD) | 9,574 (79.9) | 7,726 (80.6) | 7,778 (80.1) | 7,627 (81.1) | 7,703 (80.6) | 7,716 (80.8) |
| Citalopram  N (% of SSRI) | 7,247 (46.3) | 5,924 (46.6) | 5,951 (46.6) | 5,831(46.7) | 5,839 (46.7) | 5,839 (46.7) |
| Mirtazapine  N (% of Other class) | 1,750 (46.6) | 1,321 (45.0) | 1,334 (45.0) | 1,297 (45.4) | 1,298 (45.5) | 1,298 (45.5) |
| 10-year | | | | | | |
| Antidepressant  N (%) | 9,126 (6.0) | 7,242 (6.0) | 7,449 (6.1) | 7,124 (6.0) | 7,240 (6.1) | 7,269 (6.1) |
| SSRI  N (% of AD) | 7,379 (80.9) | 5,941 (82.0) | 6,121 (82.2) | 5,824 (81.2) | 5,941 (82.1) | 5,931 (81.6) |
| Citalopram  N (% of SSRI) | 7,175 (46.5) | 5,865 (46.7) | 5,892 (46.7) | 5,771 (46.8) | 5,779 (46.8) | 5,779 (46.8) |
| Mirtazapine  N (% of Other class) | 1,673 (46.2) | 1,258 (44.5) | 1,272 (44.6) | 1,234 (44.9) | 1,234 (44.9) | 1,234 (44.9) |

Appendix 3a: List of confounders included in the models looking at the exposure of any antidepressant

| Follow-up period | Outcome | Confounders selected by model |
| --- | --- | --- |
| *5-year* | | |
|  | | |
|  | Diabetes | Age^$^, gender, HbA1c^$***^, BMI^***^, WHR^$***^, triglycerides^***^, long-term illness^**^, smoking status, parental diabetes history^**^, benefits status^**^, urban/rural status^***^, household income^$^ |
|  | Hypertension | Age^$^***, gender**, BMI***, WHR**, triglycerides***, vitamin D^$^***, parental hypertension history***, alcohol intake status^$^, urban/rural status*** |
|  | CVA | Age^$^, gender**, accommodation status^$^**, benefits status*** |
|  | CHD | Age^$^***, gender***, apolipoprotein A***, apolipoprotein B***, vitamin D**, long-term illness***, parental CHD history*, education^$^*** |
|  | CVD mortality | Age^$*^, gender**, WHR***, employment status^$^***, physical activity^$^ |
|  | ACM | Age^$^*, gender*, BMI^$^, WHR***, long-term illness***, benefits status***, household income^$^** |
| *10-year* | | |
|  | | |
|  | Diabetes | Age^$^, gender*, apolipoprotein A***, HbA1c^$^***, BMI***, WHR^$^***, triglycerides***, vitamin D***, long-term illness**, parental diabetes history***, benefits status***, urban/rural status***, household income^$^ |
|  | Hypertension | Age^$^***, gender***, BMI***, WHR***, triglycerides***, vitamin D^$^***, parental hypertension history***, benefits status, urban/rural status***, household income^$^* |
|  | CVA | Age^$^**, gender***, vitamin D***, long-term illness**, parental CVA history**, number of vehicles per household^$^***, benefits status*** |
|  | CHD | Age^$^***, gender***, apolipoprotein A***, apolipoprotein B***, BMI**, vitamin D, long-term illness***, parental CHD history, employment status^$^*, education^$^*** |
|  | CVD mortality | Age^$^*, gender***, HbA1c***, WHR***, long-term illness***, smoking status**, number of vehicles per household^$^***, employment status^$^**, benefits status**, physical activity^$^ |
|  | ACM | Age^$^***, gender***, HbA1c**, BMI^$^***, WHR***, vitamin D**, long-term illness***, smoking status***, number of vehicles per household^$^***, employment status^$^**, benefits status***, education^$^*, physical activity^$*^ |

^$^non-linear term

*p<0.05, **p<0.01, ***p<0.001

Appendix 3b: List of confounders included in the models looking at the exposure of SSRI treatment

| Follow-up period | Outcome | Confounders selected by model |
| --- | --- | --- |
| *5-year* | | |
|  | | |
| SSRI | Diabetes | Age^$^, gender*, HbA1c^$^***, BMI***, WHR^$^***, triglycerides, long-term illness**, parental diabetes history***, benefits status**, urban/rural status*** |
|  | Hypertension | Age^$^***, gender**, BMI***, WHR**, triglycerides***, vitamin D^$^***, parental hypertension history***, alcohol intake status, urban/rural status^$^*** |
|  | CVA | Age^$^, gender**, long-term illness***, accommodation status^$^** |
|  | CHD | Age^$^**, gender***, apolipoprotein A***, apolipoprotein B***, WHR**, vitamin D*, long-term illness**, parental CHD history, education^$^*** |
|  | CVD mortality | Age^$^, gender**, WHR***, employment status^$^***, physical activity^$^ |
|  | ACM | Age^$*^, gender**, WHR***, long-term illness***, number of vehicles per household^$^***, benefits status***, urban/rural status, household income^$^ |
| *10-year* | | |
|  | | |
| SSRI | Diabetes | Age^$^, gender*, apolipoprotein A***, HbA1c^$^***, BMI^$^***, WHR^$^***, triglycerides***, vitamin D***, long-term illness**, smoking status, parental diabetes history***, benefits status***, urban/rural status***, household income^$^ |
|  | Hypertension | Age^$^***, gender***, BMI***, WHR***, triglycerides***, vitamin D^$^***, parental hypertension history***, benefits status, urban/rural status***, household income^$^ |
|  | CVA | Age^$^**, gender***, vitamin D***, long-term illness**, parental CVA history**, number of vehicles per household^$^**, benefits status*** |
|  | CHD | Age^$^***, gender***, apolipoprotein A***, apolipoprotein B***, BMI**, vitamin D***, long-term illness***, parental CHD history, employment status^$^*, education^$^*** |
|  | CVD mortality | Age^$*^, gender***, HbA1c***, WHR***, long-term illness***, smoking status*, number of vehicles per household^$^***, employment status^$^**, benefits status**, physical activity^$*^ |
|  | ACM | Age^$^***, gender***, HbA1c***, BMI^$^***, WHR***, vitamin D**, long-term illness***, smoking status***, number of vehicles per household^$^***, employment status^$^, benefits status***, education^$^, physical activity^$*^ |

^$^non-linear term

*p<0.05, **p<0.01, ***p<0.001

Appendix 3c: List of confounders included in the models looking at the exposure of ‘Other’ class of antidepressants

| Follow-up period | Outcome | Confounders selected by model |
| --- | --- | --- |
| *5-year* | | |
|  | | |
| Other | Diabetes | Age^$^, gender**, HbA1c^$^***, BMI***, WHR^$^***, triglycerides***, long-term illness**, parental diabetes history***, benefits status**, urban/rural status*** |
|  | Hypertension | Age^$^***, gender, BMI***, WHR***, triglycerides***, vitamin D***, parental hypertension history***, urban/rural status*** |
|  | CVA | Age^$^, gender**, accommodation status^$^** |
|  | CHD | Age^$^**, gender***, apolipoprotein A***, apolipoprotein B***, HbA1c***, long-term illness***, parental CHD history, employment status^$^, education^$^ |
|  | CVD mortality | Age^$^*, gender**, WHR***, employment status^$^** |
|  | ACM | Age^$^***, gender*, BMI^$^***, WHR***, long-term illness***, smoking status***, number of vehicles per household^$^***, benefits status*** |
| *10-year* | | |
|  | | |
| Other | Diabetes | Age^$^*, gender, apolipoprotein A***, HbA1c^$^***, BMI***, WHR^$^***, triglycerides***, long-term illness**, smoking status, parental diabetes history***, benefits status***, urban/rural status*** |
|  | Hypertension | Age^$^***, gender***, BMI***, WHR***, triglycerides***, vitamin D***, parental hypertension history***, urban/rural status***, household income^$^ |
|  | CVA | Age^$^**, gender***, vitamin D***, long-term illness***, parental CVA history**, accommodation status^$^, number of vehicles per household^$^ |
|  | CHD | Age^$^***, gender***, apolipoprotein A***, apolipoprotein B***, HbA1c**, BMI***, vitamin D***, long-term illness***, parental CHD history, education^$^ |
|  | CVD mortality | Age^$^, gender***, HbA1c***, WHR***, long-term illness**, smoking status*, number of vehicles per household^$^**, employment status^$^, benefits status***, physical activity^$^ |
|  | ACM | Age^$^***, gender***, HbA1c**, BMI^$^***, WHR***, vitamin D**, long-term illness***, smoking status***, number of vehicles per household^$^***, employment status^$^, benefits status***, education^$^ |

^$^non-linear term

*p<0.05, **p<0.01, ***p<0.001

Appendix 4a: Kaplan Meier curves at 5-year follow up


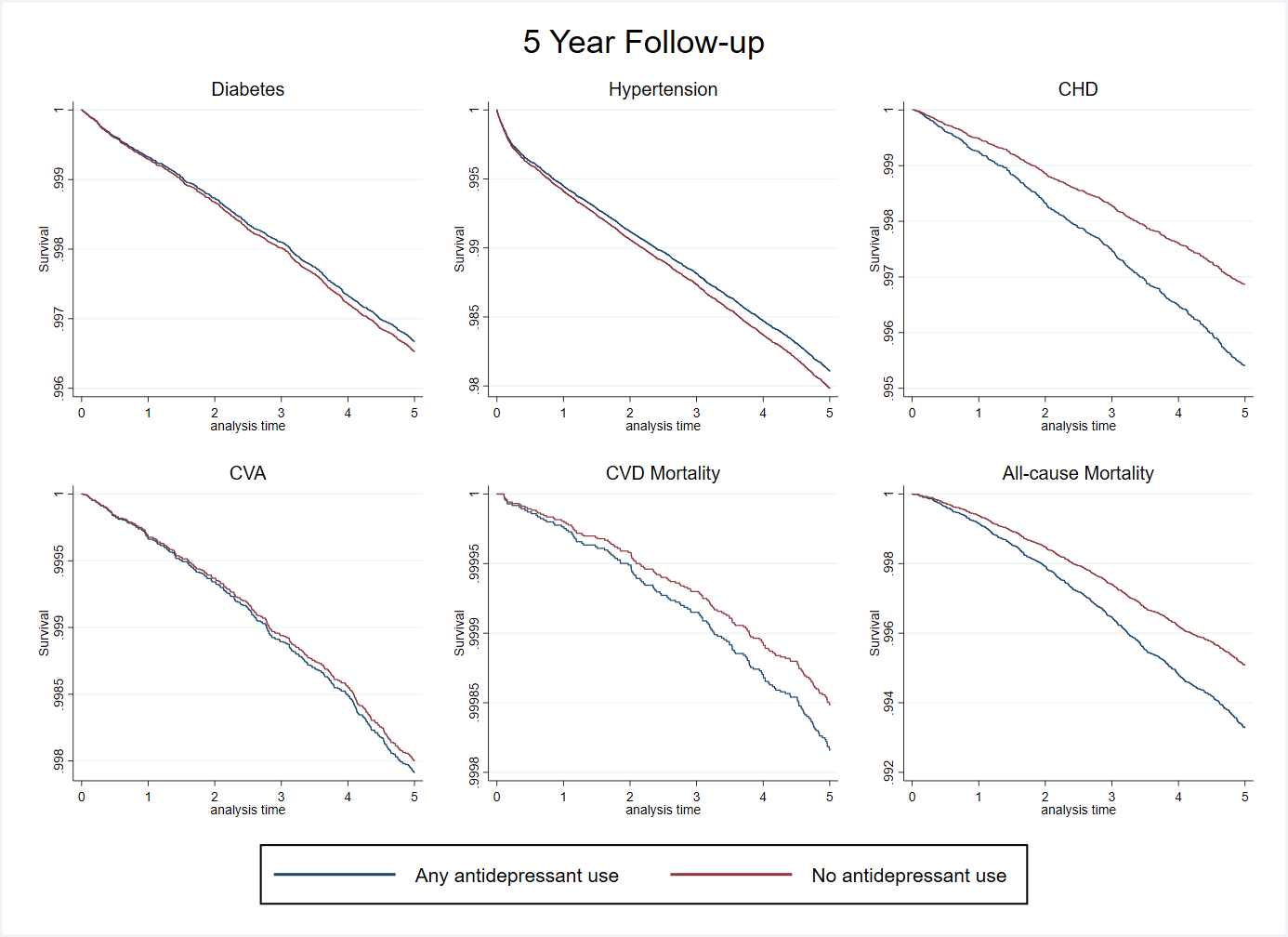


Appendix 4b: Kaplan Meier curves at 10-year follow up


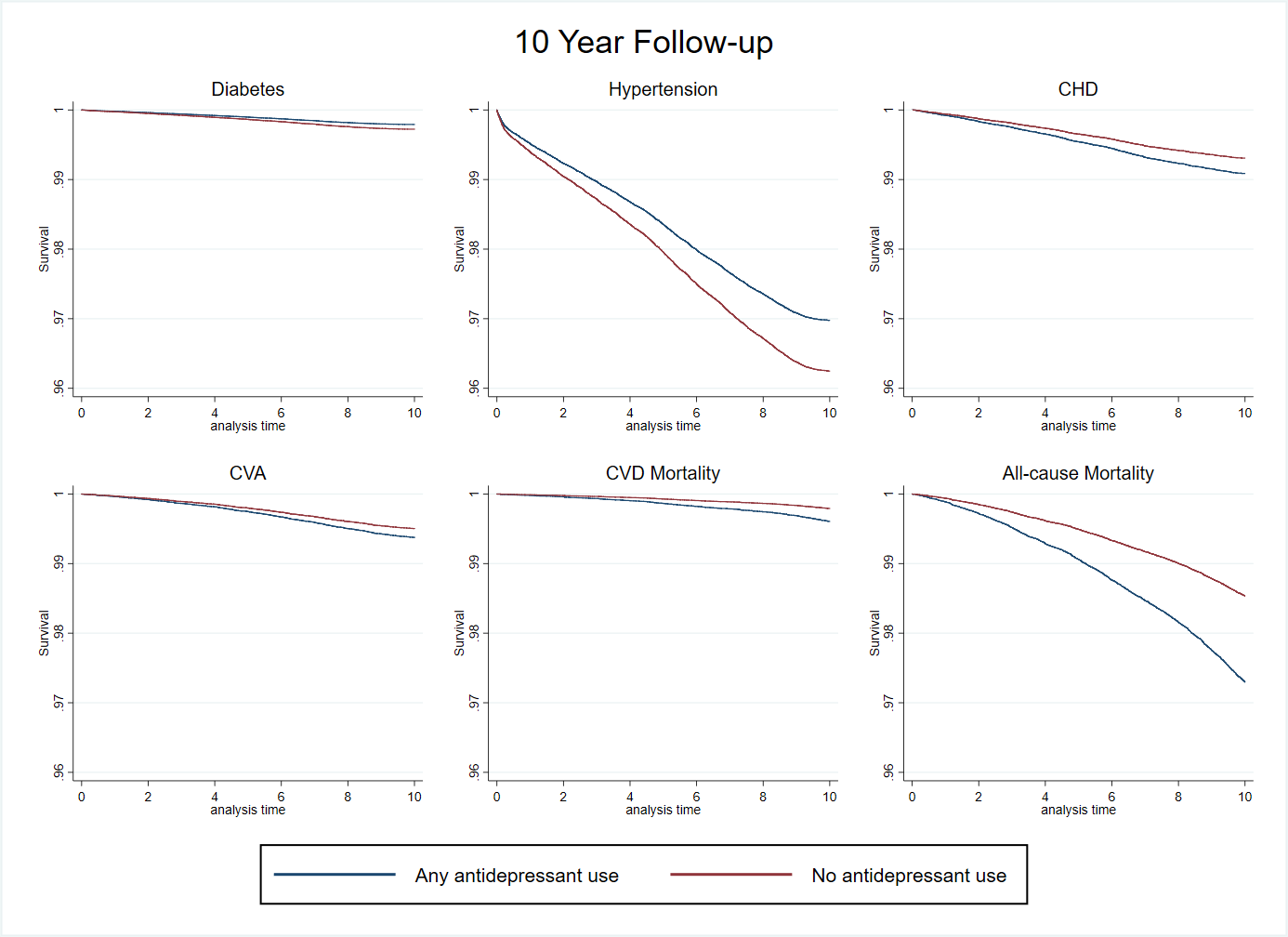


Appendix 5: Sensitivity analysis excluding short term antidepressant use (<90 days)

|  | Participants | Observations | Events | Person years | HR | 95% CI |
| --- | --- | --- | --- | --- | --- | --- |
| *5-year follow-up* |  |  |  |  |  |  |
| Diabetes | 112,207 | 120,582 | 950 | 537,018 |  |  |
| No AD |  |  | 865 | 491,174 | 1.00 |  |
| Any AD |  |  | 85 | 45,843 | 0.83 | (0.67-1.06) |
|  |  |  |  |  |  |  |
| Hypertension | 105,440 | 113,375 | 2,584 | 500,154 |  |  |
| No AD |  |  | 2394 | 456,496 | 1.00 |  |
| Any AD |  |  | 190 | 43,657 | 0.91 | (0.79-1.06) |
|  |  |  |  |  |  |  |
| CVA | 119,069 | 128,165 | 336 | 571,681 |  |  |
| No AD |  |  | 306 | 521465 | 1.00 |  |
| Any AD |  |  | 30 | 50,215 | 1.10 | (0.75-1.60) |
|  |  |  |  |  |  |  |
| CHD | 79,596 | 85,304 | 538 | 382,139 |  |  |
| No AD |  |  | 488 | 350,725 | 1.00 |  |
| Any AD |  |  | 50 | 31,413 | 1.52 | (1.13-2.05) |
|  |  |  |  |  |  |  |
| CVD mortality | 111,840 | 120,250 | 147 | 537,761 |  |  |
| No AD |  |  | 135 | 491,290 | 1.00 |  |
| Any AD |  |  | 12 | 46,471 | 1.04 | (0.57-1.90) |
|  |  |  |  |  |  |  |
| All-cause mortality | 99,894 | 107,426 | 703 | 480,230 |  |  |
| No AD |  |  | 645 | 438,834 | 1.00 |  |
| Any AD |  |  | 58 | 41,396 | 0.87 | (0.66-1.14) |
|  |  |  |  |  |  |  |
| *10-year follow-up* |  |  |  |  |  |  |
| Diabetes | 95,576 | 104,342 | 1,422 | 865,671 |  |  |
| No AD |  |  | 1,327 | 806,344 | 1.00 |  |
| Any AD |  |  | 95 | 59,327 | 0.83 | (0.67-1.03) |
|  |  |  |  |  |  |  |
| Hypertension | 89,765 | 98,112 | 3,688 | 800,999 |  |  |
| No AD |  |  | 3,497 | 744,779 | 1.00 |  |
| Any AD |  |  | 191 | 56,220 | 0.79 | (0.68-0.92) |
|  |  |  |  |  |  |  |
| CVA | 102,353 | 111,864 | 674 | 933,859 |  |  |
| No AD |  |  | 620 | 869,053 | 1.00 |  |
| Any AD |  |  | 54 | 64,805 | 1.23 | (0.92-1.63) |
|  |  |  |  |  |  |  |
| CHD | 77,613 | 84,532 | 990 | 707,320 |  |  |
| No AD |  |  | 921 | 660,150 | 1.00 |  |
| Any AD |  |  | 69 | 47,170 | 1.37 | (1.07-1.76) |
|  |  |  |  |  |  |  |
| CVD mortality | 100,533 | 109,845 | 434 | 920,051 |  |  |
| No AD |  |  | 388 | 856,516 | 1.00 |  |
| Any AD |  |  | 46 | 63,534 | 1.61 | (1.17-2.21) |
|  |  |  |  |  |  |  |
| All-cause mortality | 78,953 | 85,995 | 1,671 | 724,214 |  |  |
| No AD |  |  | 1505 | 676,204 | 1.00 |  |
| Any AD |  |  | 166 | 48,009 | 1.54 | (1.31-1.82) |
